# Concomitant Gastric and Duodenal Wall Necrosis as a Rare Late Complication of Severe Acute Pancreatitis: A Case Report

**DOI:** 10.7759/cureus.101666

**Published:** 2026-01-16

**Authors:** Hamza Najout, Walid Atmani, Ilyass Masad, Mustapha Bensghir

**Affiliations:** 1 Department of Anesthesia and Intensive Care Unit, Mohammed V Military Teaching Hospital, Mohammed V University, Rabat, MAR

**Keywords:** duodenal necrosis, extra-pancreatic complications, gastric necrosis, ischemic necrosis, multiorgan failure, severe acute pancreatitis

## Abstract

Severe acute pancreatitis (SAP) is a life-threatening inflammatory condition that can occasionally result in rare and devastating extra-pancreatic complications. Among these, gastric or duodenal wall necrosis is exceptionally uncommon but carries a high risk of mortality.

We report the case of a 56-year-old man with idiopathic SAP who initially improved with conservative management. After systematic exclusion of common etiologies, including biliary, alcoholic, metabolic, infectious, and drug-induced causes, the pancreatitis was classified as idiopathic. Three weeks after admission, he developed recurrent abdominal pain, fever, and hemodynamic instability. Contrast-enhanced computed tomography (CECT) revealed non-enhancing posterior gastric and duodenal walls with intramural gas, indicating ischemic and infected necrosis. Despite prompt surgical debridement, intensive care management, and broad-spectrum antibiotics, the patient developed multiorgan failure and subsequently died. Histopathology confirmed ischemic necrosis involving both the gastric and duodenal walls.

This case underscores the multifactorial pathogenesis of such complications, combining microvascular thrombosis, enzymatic vascular injury, and bacterial infection. Because gastric and duodenal necrosis often occur in the late phase of pancreatitis, sometimes after apparent recovery, their diagnosis can be delayed. Therefore, any recurrence of abdominal pain or systemic instability in SAP should prompt immediate imaging to rule out delayed ischemic complications. Early recognition and multidisciplinary intervention remain essential to improve outcomes in these rare but catastrophic manifestations of severe pancreatitis.

## Introduction

Severe acute pancreatitis (SAP) is a life-threatening inflammatory disease characterized by diffuse pancreatic injury and an intense systemic inflammatory response, frequently leading to persistent organ failure and multiorgan dysfunction [1,2]. The clinical spectrum of acute pancreatitis ranges from mild, self-limiting edematous forms to severe necrotizing pancreatitis, in which local and systemic complications are the primary determinants of prognosis. Despite significant advances in critical care, imaging, and minimally invasive therapeutic strategies, SAP continues to pose a major clinical challenge, with reported mortality rates reaching 30%-50% in complicated cases [3].

Pancreatic and peripancreatic necrosis represents the hallmark local complication of SAP and may extend into surrounding tissues as a result of enzymatic autodigestion, inflammatory edema, and vascular injury [4]. In contrast, secondary involvement of adjacent hollow viscera, particularly the stomach and duodenum, remains exceedingly rare. Owing to their rich vascular supply and robust mucosal defense mechanisms, these organs are usually resistant to ischemic injury, which explains the scarcity of reported cases of gastric or duodenal wall necrosis in the setting of acute pancreatitis [5].

When present, gastrointestinal wall necrosis typically occurs during the late phase of SAP, often after an initial period of apparent clinical improvement. The underlying pathophysiological mechanisms are complex and multifactorial, involving persistent local inflammation, direct enzymatic tissue destruction, ischemic injury related to microvascular thrombosis or vascular compression, and secondary bacterial infection [6,7]. These processes may progress insidiously, making early clinical recognition particularly challenging.

The clinical presentation of such complications is often nonspecific and may mimic recurrent pancreatitis, infected pancreatic necrosis, or gastrointestinal perforation. Consequently, diagnosis is frequently delayed, contributing to increased morbidity and mortality. Contrast-enhanced computed tomography (CECT) plays a pivotal role in early detection, while histopathological analysis is essential to confirm ischemic and infected necrosis when surgical intervention is required [8].

We report a rare case of concomitant gastric and duodenal wall necrosis occurring as a late complication of idiopathic severe acute pancreatitis. By integrating clinical, radiological, and pathological findings, this case highlights the need for sustained vigilance during the post-acute phase of SAP and underscores the importance of early imaging and multidisciplinary management to improve patient outcomes.

## Case presentation

A 56-year-old man, previously healthy and with no history of alcohol consumption, metabolic disorder, or biliary disease, presented to the emergency department with abrupt-onset, severe epigastric pain radiating to the back, accompanied by persistent vomiting and fever. On examination, he appeared distressed but hemodynamically stable. Laboratory analysis revealed severe inflammatory response and pancreatic injury: white blood cell count (WBC), 18 × 10⁹/L; C-reactive protein (CRP), 210 mg/L; procalcitonin (PCT), 3.5 ng/mL; lactate dehydrogenase (LDH), 560 U/L; and serum lipase exceeding three times the upper limit of normal, confirming the diagnosis of acute pancreatitis.

**Figure 1:**
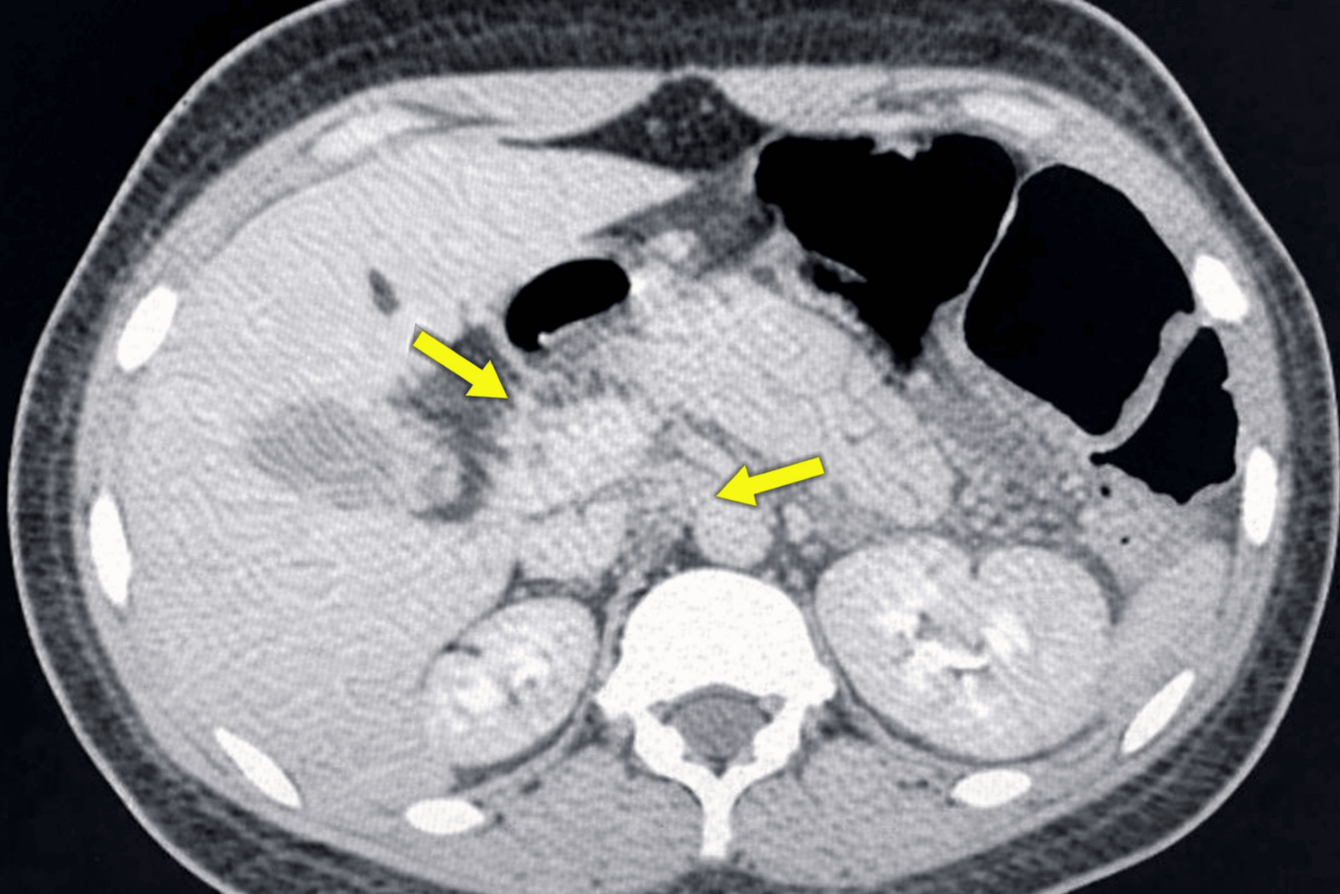
Initial contrast-enhanced computed tomography findings of severe acute pancreatitis Axial contrast-enhanced computed tomography of the abdomen demonstrating diffuse enlargement of the pancreas with ill-defined margins and surrounding peripancreatic fat stranding (yellow arrows), consistent with edematous severe acute pancreatitis at presentation.

Initial contrast-enhanced computed tomography (CECT) of the abdomen demonstrated diffuse pancreatic enlargement with blurred margins and peripancreatic fat stranding, findings characteristic of severe acute pancreatitis (Figure 1).

No gallstones, biliary dilatation, or ductal obstruction were identified. The etiologic evaluation, including metabolic tests (serum triglycerides and calcium), autoimmune screening (IgG4), and biliary imaging, was unremarkable, establishing an idiopathic etiology.

The patient was admitted to the intensive care unit and managed conservatively with aggressive crystalloid resuscitation, multimodal analgesia, early enteral nutrition, and close hemodynamic and metabolic monitoring. Over the following two weeks, progressive clinical and biochemical improvement was observed with normalization of inflammatory markers (WBC: 9 × 10⁹/L, CRP: 45 mg/L, PCT: 0.4 ng/mL, LDH: 290 U/L).

**Figure 2:**
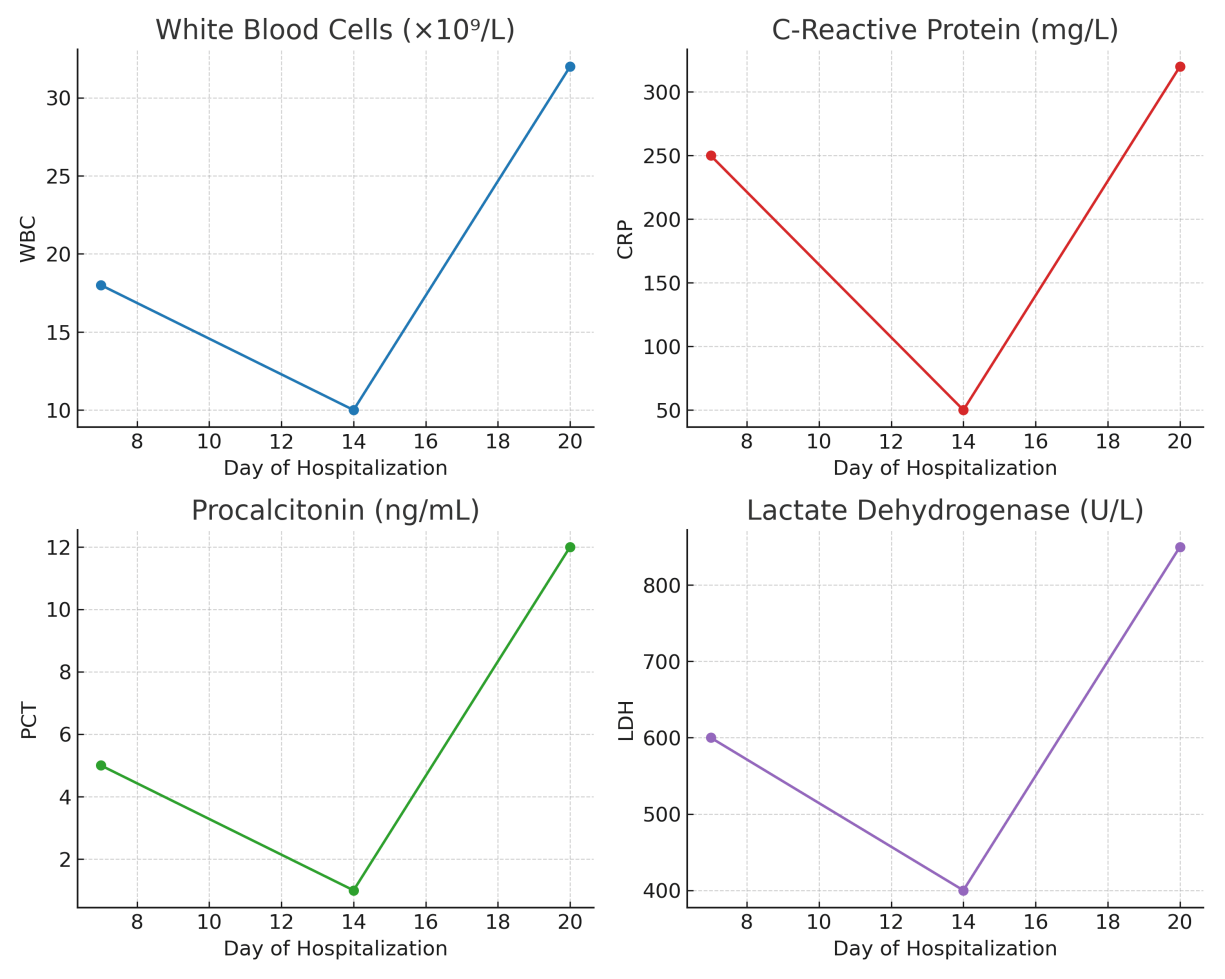
Temporal evolution of inflammatory biomarkers during hospitalization WBC, CRP, PCT, and LDH initially decreased under conservative management, followed by a marked secondary inflammatory rebound around day 20, coinciding with clinical deterioration. WBC: white blood cell count, CRP: C-reactive protein, PCT: procalcitonin, LDH: lactate dehydrogenase

On the 20th day of hospitalization, the patient experienced a sudden recrudescence of epigastric pain associated with high-grade fever and hypotension refractory to fluid resuscitation. Laboratory reassessment revealed marked secondary deterioration (WBC: 31 × 10⁹/L, CRP: 320 mg/L, PCT: 12 ng/mL, LDH: 870 U/L) (Figure 2).

**Figure 3:**
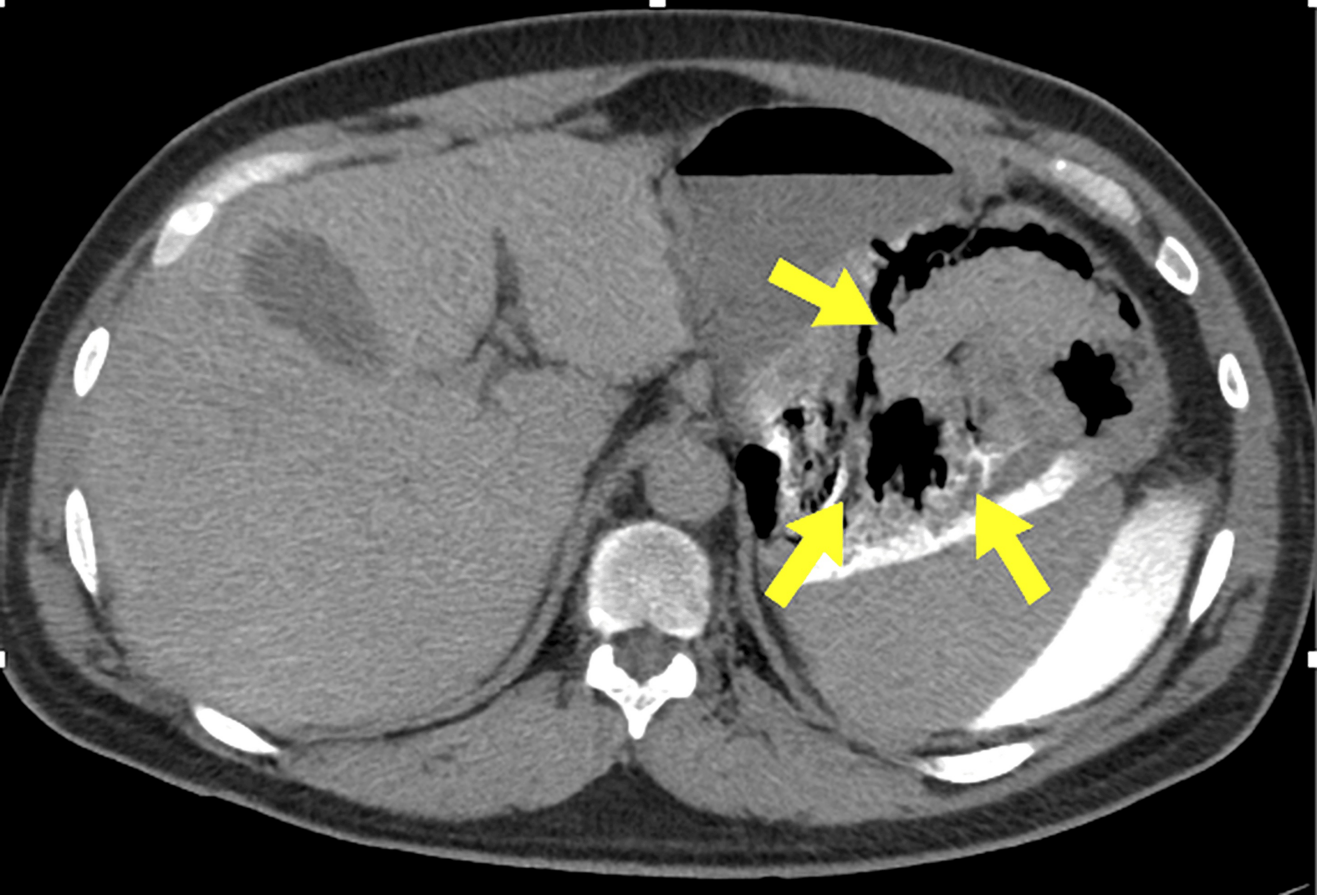
Contrast-enhanced CT findings of late gastrointestinal necrosis Axial contrast-enhanced CT of the abdomen demonstrating non-enhancing thickening of the posterior gastric wall and the adjacent duodenal wall (yellow arrows), associated with intramural gas and surrounding inflammatory infiltration, consistent with ischemic and infected necrosis as a late complication of severe acute pancreatitis. CT: computed tomography

A repeat contrast-enhanced CT demonstrated extensive non-enhancing thickening of the posterior gastric wall and duodenal frame with multiple intramural gas foci and surrounding inflammatory infiltration, highly suggestive of ischemic and infected necrosis involving both organs (Figure 3).

Following a multidisciplinary discussion, a conservative operative strategy was adopted, focusing on necrotic tissue debridement while attempting to maintain gastrointestinal continuity. The patient underwent emergency surgery. Intraoperatively, the posterior gastric wall and the first portion of the duodenum were friable, gray-black in color, and surrounded by purulent collections within the lesser sac. Extensive debridement of necrotic gastric and duodenal tissues was performed with the aim of achieving adequate source control while preserving gastrointestinal continuity. Multiple large-bore drains were placed within the lesser sac and perigastric region for continuous postoperative drainage. Gastric decompression was ensured via nasogastric tube. Due to profound septic shock, severe hemodynamic instability, and extremely high operative risk, no formal gastric or biliary diversion procedures and no feeding jejunostomy were undertaken. Postoperative nutritional support was therefore provided exclusively through parenteral nutrition.

**Figure 4:**
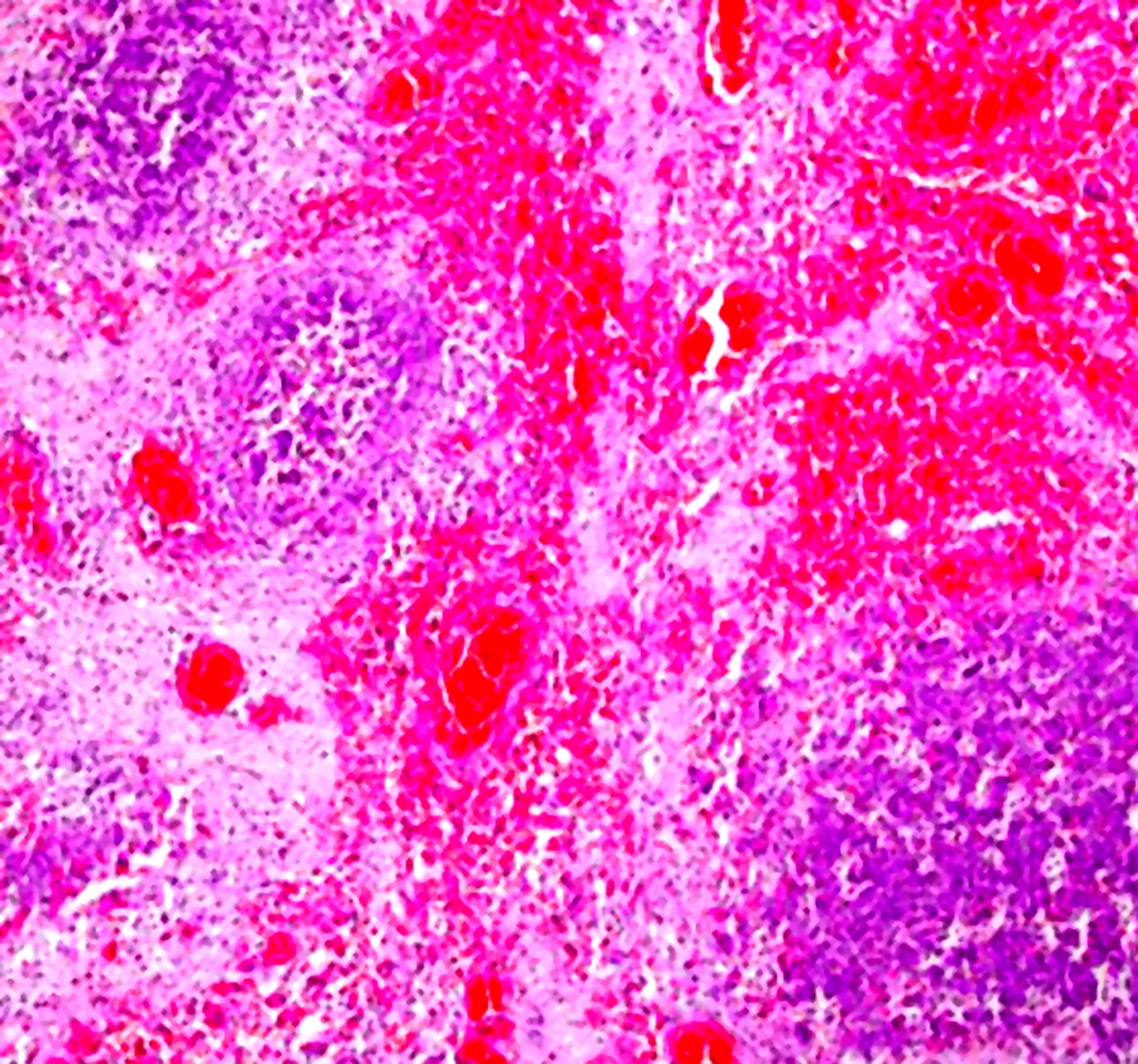
Histopathological findings of gastrointestinal wall necrosis Histopathological examination of the resected gastrointestinal wall (hematoxylin and eosin staining) showing extensive transmural coagulative necrosis with loss of normal tissue architecture, associated with vascular thrombosis and dense inflammatory infiltration. These findings confirm ischemic and infected necrosis of the gastric and duodenal walls.

Histopathological analysis of resected specimens demonstrated transmural coagulative necrosis with vascular thrombosis and dense inflammatory infiltration by Gram-negative bacilli, confirming ischemic and infected necrosis of both gastric and duodenal walls (Figure 4).

Despite aggressive postoperative resuscitation, broad-spectrum antibiotic therapy, and full organ-supportive measures, the patient’s condition progressively deteriorated. He developed refractory septic shock and multiorgan failure, culminating in death on day 35 of hospitalization.

## Discussion

To our knowledge, only a very limited number of cases have described concomitant gastric and duodenal wall necrosis as a delayed complication of severe acute pancreatitis (SAP). This exceptionally rare manifestation reflects the profound local inflammatory and vascular disturbances that characterize the most severe forms of the disease. Notably, gastric or duodenal necrosis typically arises during the late phase of pancreatitis, often after an initial period of apparent clinical improvement, which may lead to a false sense of resolution and delay both diagnosis and intervention [2].

The pathophysiology underlying this complication is complex and multifactorial. During SAP, premature activation of pancreatic enzymes, including trypsin, elastase, and phospholipase A₂, triggers peripancreatic fat necrosis and progressive endothelial injury. This vascular damage promotes microcirculatory impairment, microvascular thrombosis, and regional ischemia, ultimately resulting in transmural necrosis of adjacent structures [4]. The posterior gastric wall and the duodenum are particularly vulnerable because of their close anatomical relationship to the pancreas and their shared arterial supply via branches of the splenic and gastroduodenal arteries. In addition, external compression by peripancreatic collections or walled-off necrosis can further compromise local perfusion, as likely occurred in our patient.

Microcirculatory dysfunction represents a central mechanism in the progression toward gastrointestinal wall necrosis. Reduced splanchnic blood flow, capillary leakage, endothelial swelling, and increased intra-abdominal pressure collectively lead to tissue hypoxia. Subsequent reperfusion may exacerbate injury through oxidative stress and inflammatory mediator release, culminating in irreversible cellular necrosis [3,9]. In our case, the presence of intramural gas on imaging strongly suggested bacterial superinfection, a feature associated with a particularly poor prognosis. Infected necrosis is most commonly caused by Gram-negative organisms belonging to the Enterobacteriaceae family, although culture results may be negative due to prior antibiotic therapy or sampling limitations [10].

Clinically, this entity is difficult to recognize, as it may mimic other intra-abdominal emergencies such as perforated peptic ulcer, ischemic gastritis, or stress-related mucosal injury. Consequently, contrast-enhanced computed tomography remains indispensable for diagnosis, as it allows accurate assessment of both the extent and nature of the necrotic process [5,11]. Typical radiological findings include non-enhancing gastric or duodenal wall thickening, surrounding inflammatory infiltration, and intramural gas. Upper gastrointestinal endoscopy may reveal black or gray necrotic mucosa but is often avoided in unstable patients because of the significant risk of perforation.

Therapeutic management is particularly challenging, and no standardized treatment algorithm exists due to the extreme rarity of this complication. Optimal outcomes rely on early recognition and a coordinated multidisciplinary approach involving anesthesiologists, surgeons, gastroenterologists, and radiologists. Minimally invasive “step-up” strategies, starting with percutaneous or endoscopic drainage. are generally preferred when feasible, as they are associated with reduced morbidity in infected pancreatic necrosis [12]. However, in cases of extensive gastric or duodenal necrosis complicated by uncontrolled sepsis or impending perforation, surgical debridement remains essential to achieve definitive source control, as was necessary in our patient.

The prognosis of concomitant gastric and duodenal necrosis remains poor, with mortality largely driven by septic shock and multiorgan failure despite aggressive supportive care. As illustrated by this case, recurrence of abdominal pain, hemodynamic instability, and secondary elevation of inflammatory markers after an initial phase of improvement should immediately raise suspicion for evolving extra-pancreatic necrosis. Early repeat imaging is therefore critical to guide timely intervention.

Overall, this case underscores the importance of sustained clinical vigilance during the convalescent phase of severe acute pancreatitis. Apparent clinical improvement should not preclude close monitoring, as delayed ischemic and infectious complications may develop insidiously. Maintaining a high index of suspicion, performing prompt radiological reassessment, and ensuring early multidisciplinary involvement are essential to improve outcomes in this rare but devastating condition [8].

## Conclusions

Concomitant gastric and duodenal wall necrosis represents an exceptionally rare and devastating late manifestation of severe acute pancreatitis. This case illustrates the unpredictable clinical course of pancreatitis and the potential for catastrophic extra-pancreatic complications even after an apparent phase of recovery. Early recognition through vigilant clinical monitoring and timely contrast-enhanced imaging is essential, particularly in patients presenting with recurrent abdominal pain, secondary inflammatory elevation, or hemodynamic instability during the convalescent phase. A multidisciplinary approach involving anesthesiologists, gastroenterologists, surgeons, and radiologists is crucial to ensure accurate diagnosis, appropriate timing of intervention, and coordinated perioperative care. Management must be individualized, balancing minimally invasive strategies with surgical debridement in the presence of extensive necrosis or sepsis. Ultimately, this case underscores that clinical improvement should not be equated with disease resolution, and sustained surveillance remains vital to detect delayed ischemic and infectious complications and improve patient outcomes.
